# Differentially Expressed Genes in Response to a Squalene-Supplemented Diet Are Accurate Discriminants of Porcine Non-Alcoholic Steatohepatitis

**DOI:** 10.3390/ijms241612552

**Published:** 2023-08-08

**Authors:** Roubi Abuobeid, Luis V. Herrera-Marcos, Carmen Arnal, Seyed Hesamoddin Bidooki, Javier Sánchez-Marco, Roberto Lasheras, Joaquín C. Surra, María Jesús Rodríguez-Yoldi, Roberto Martínez-Beamonte, Jesús Osada

**Affiliations:** 1 Departamento de Bioquímica y Biología Molecular y Celular, Facultad de Veterinaria, Instituto de Investigación Sanitaria de Aragón, Universidad de Zaragoza, E-50013 Zaragoza, Spain; 2 Instituto Agroalimentario de Aragón, CITA-Universidad de Zaragoza, E-50013 Zaragoza, Spain; 3 Departamento de Patología Animal, Facultad de Veterinaria, Instituto de Investigación Sanitaria de Aragón, Universidad de Zaragoza, E-50013 Zaragoza, Spain; 4 CIBER de Fisiopatología de la Obesidad y Nutrición, Instituto de Salud Carlos III, E-28029 Madrid, Spain; 5 Laboratorio Agroambiental, Servicio de Seguridad Agroalimentaria de la Dirección General de Alimentación y Fomento Agroalimentario, Gobierno de Aragón, E-50071 Zaragoza, Spain; 6 Departamento de Producción Animal y Ciencia de los Alimentos, Escuela Politécnica Superior de Huesca, Instituto de Investigación Sanitaria de Aragón, Universidad de Zaragoza, E-22071 Huesca, Spain; 7 Departamento de Farmacología, Fisiología, Medicina Legal y Forense, Facultad de Veterinaria, Instituto de Investigación Sanitaria de Aragón, Universidad de Zaragoza, E-50013 Zaragoza, Spain

**Keywords:** squalene, virgin olive oil, pigs, murine AML12 cell line, human HEPG2 cell line, transcriptome, hepatic, RNA sequencing, *AFP*, *ENPEP*, *PPP1R1B*

## Abstract

Squalene is the major unsaponifiable component of virgin olive oil, the fat source of the Mediterranean diet. To evaluate its effect on the hepatic transcriptome, RNA sequencing was carried out in two groups of male Large White x Landrace pigs developing nonalcoholic steatohepatitis by feeding them a high fat/cholesterol/fructose and methionine and choline-deficient steatotic diet or the same diet with 0.5% squalene. Hepatic lipids, squalene content, steatosis, activity (ballooning + inflammation), and SAF (steatosis + activity + fibrosis) scores were analyzed. Pigs receiving the latter diet showed hepatic squalene accumulation and twelve significantly differentially expressed hepatic genes (log_2_ fold change < 1.5 or <1.5) correlating in a gene network. These pigs also had lower hepatic triglycerides and lipid droplet areas and higher cellular ballooning. Glutamyl aminopeptidase (*ENPEP*) was correlated with triglyceride content, while alpha-fetoprotein (*AFP*), neutralized E3 ubiquitin protein ligase 3 (*NEURL3*), 2′-5′-oligoadenylate synthase-like protein (*OASL*), and protein phosphatase 1 regulatory inhibitor subunit 1B (*PPP1R1B*) were correlated with activity reflecting inflammation and ballooning, and *NEURL3* with the SAF score. *AFP*, *ENPEP*, and *PPP1R1B* exhibited a remarkably strong discriminant power compared to those pathological parameters in both experimental groups. Moreover, the expression of *PPP1R1B*, *TMEM45B*, *AFP*, and *ENPEP* followed the same pattern in vitro using human hepatoma (HEPG2) and mouse liver 12 (AML12) cell lines incubated with squalene, indicating a direct effect of squalene on these expressions. These findings suggest that squalene accumulated in the liver is able to modulate gene expression changes that may influence the progression of non-alcoholic steatohepatitis.

## 1. Introduction

Nonalcoholic fatty liver disease (NAFLD), characterized by an imbalance between hepatic lipid output and input resulting in lipid droplet deposition [1], is the most common chronic liver metabolic disorder, with an estimated prevalence range of 6–35% worldwide and 25–26% in Europe [2]. Between 15% and 20% [3] or 15.9% and 68.3% [4] of NAFLD patients progress to nonalcoholic steatohepatitis (NASH). NASH, an inflammatory disease, is characterized by a combination of three histological features: fat accumulation or steatosis, ballooning of hepatocytes, and lobular inflammation, with or without fibrosis [5,6]. Approximately 20% of patients with chronic NASH develop cirrhosis [7] and about 85% of cirrhosis patients evolve to hepatocellular carcinoma (HCC) [8], leading to 12 times more HCC than in NAFLD [9]. In this metabolic disorder, hepatic gene transcriptome changes have been found to be involved [10,11].

Since the 1960s, the Mediterranean diet has been recognized as a healthy choice against major chronic degenerative diseases [12,13]. Virgin olive oil (VOO) is the main fat component in this diet [14]. VOO is not only composed of triglycerides containing monounsaturated fatty acids, but also of minor bioactive phytochemicals that have important health benefits [15]. The terpene squalene is one of the most abundant minor components present in a wide range (2.4 to 9.3 g/kg of VOO) depending on cultivar, climate, and ripeness of olive fruit [16]. This hydrocarbon is a precursor in the cholesterol biosynthesis [17], and its administration has been shown to exert anti-neoplastic, anti-inflammatory, and antioxidant properties, and to control glucose and lipid metabolisms [18,19].

Squalene received its name because it was first discovered in the livers of sharks [20]. Some species belonging to the family Squalidae can accumulate this compound in their livers up to 50–82% of their liver weight [21]. The lifespan of these sharks is currently unknown. Other sharks, such as *Somniosus microcephalus*, are known for having one of the longest lifespans among vertebrates (392 ± 120 years) [22]. However, the amount of squalene in their livers has not been measured [23]. In this way, the impact of squalene on hepatic content and lifespan remains uncertain. Thus, the effect of squalene on NASH pathogenesis also requires further investigation. Kritchevsky et al. [24] were the first to prove that dietary squalene accumulated in the rabbit liver. Recent studies, utilizing mouse and rabbit models, have shown distinct subcellular accumulation depending on the experimental model [25]. The first model accumulated squalene in large lipid droplets and smooth reticulum fractions, whereas the second did so in small-size vesicles and the rough endoplasmic reticulum. Furthermore, squalene modulated hepatic lipids, particularly triglycerides and cholesterol, with different outcomes in both models. In mice, hepatic triglyceride content was reduced, whereas this effect was not observed in rabbits [19,26]. Squalene administration induced important changes in hepatic proteins involved in lipid metabolism, oxidative stress, and lipoprotein secretion, which could explain the observed reduction in hepatic triglycerides in mice [27]. The accumulation of squalene in the liver of mice was associated with complex transcriptional and post-translational changes in gene expressions, including of *Mat1*, *Acox1*, *Casp1*, *Cyp2b10*, *Cyp2c55*, *Cpt1a*, and *Txndc5* [27,28,29]. In contrast, the hepatic changes in rabbit involved *PNPLA3*, *GCK*, *TFCP2L1*, *ASCL1*, *ACSS2*, *OST4*, *FAM91A1*, *MYH6*, *LRRC39*, *LOC108176846*, *GLT1D1*, and *TREH* [30]. These discrepant findings in animal models need further confirmation in different models and might indicate varied metabolic responses in humans. In this regard, the swine model represents an interesting approach due to its metabolic and physiological similarities to humans [31]. Our group has recently developed a model of porcine NASH using a dietary manipulation [32], and squalene has been shown to improve steatosis in this model despite an increase in the ballooning score. Furthermore, there were no changes in inflammation or fibrosis [33]. This experimental design provides an interesting approach to discover specific gene expression changes involved in controlling ballooning and steatosis. The current study was designed to identify the responsible molecular candidates through high-throughput RNA sequencing.

## 2. Results

### 2.1. Hepatic Histological Analyses

**Figure 1:**
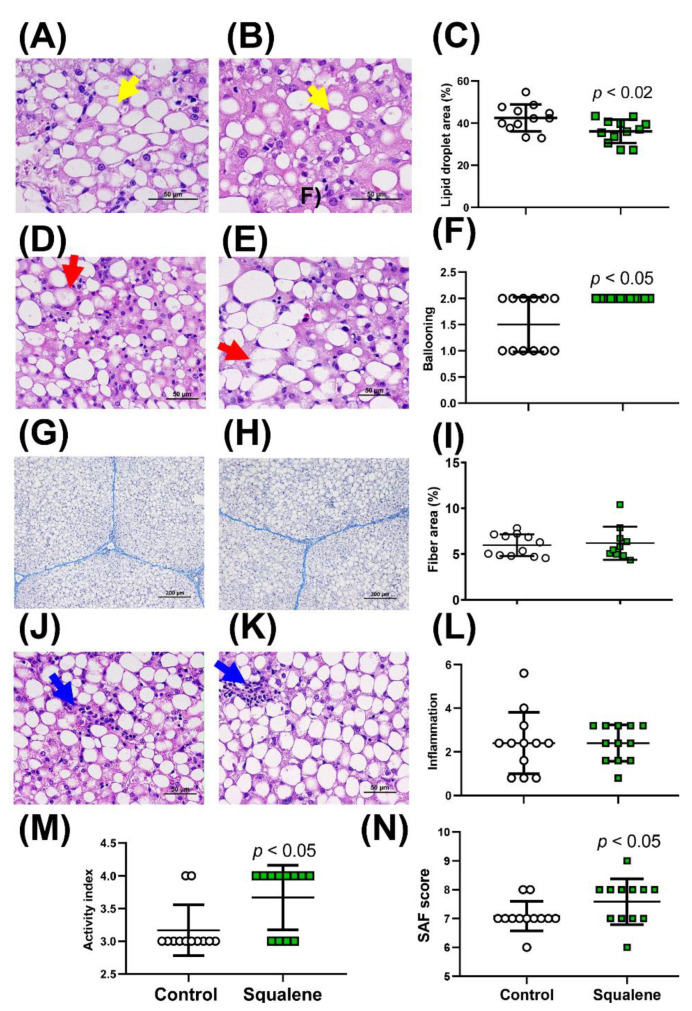
Histological analyses of male swine livers fed different diets. Representative hepatic micrographs of hematoxylin-eosin-stained sections (4 μm) from control (**A**) and squalene (**B**) groups. Bar denotes 50 μm. Morphometric changes in lipid droplet area expressed as percentage of total liver section (**C**). Selected fields displaying ballooned hepatocytes from control (**D**) and squalene (**E**) groups and quantification of ballooning (**F**). Bar denotes 50 μm. Representative liver micrographs of Masson’s trichromic-stained slides from control (**G**) and squalene groups (**H**). The scale bar represents 200 μm. Changes in fiber area (**I**) were assessed morphometrically and are expressed as a percentage of total liver section. Selected fields displaying inflammatory foci in control (**J**) and squalene (**K**) groups and quantification in all animals (**L**). Bar denotes 50 μm. The activity index (ballooning and inflammation) (**M**). The SAF score (steatosis grading, ballooning, inflammation, and fibrosis) (**N**). Lipid droplets, ballooned hepatocytes, and inflammatory foci are represented by yellow, red, and blue arrows, respectively. Data are individual results and their means and standard deviations of 12 pigs per group. Statistical analyses were performed according to the one-tailed Mann–Whitney U-test.

Pigs receiving both diets developed hepatic steatosis, as depicted in Figure 1A,B. The pigs receiving the squalene diet showed significantly decreased lipid droplet areas (Figure 1C). Likewise, the animals receiving squalene displayed a higher number of ballooned hepatocytes than the control group (Figure 1D and Figure 1E respectively), with all animals reaching a value of 2 (Figure 1F). No significant changes were observed in fibrosis (Figure 1G–I) or in the presence of inflammatory foci by squalene administration (Figure 1J–L). When the latter parameter was considered together with ballooning as the activity index [5], the group of animals receiving squalene showed a significant increase (Figure 1M) due to the increase in the ballooning score. According to the fatty liver inhibition of progression (FLIP) algorithm [5], all the animals exhibited NASH. When using a semi-quantification of steatosis, activity, and fibrosis as the SAF score [5], the pigs consuming squalene showed a significantly increased SAF score (Figure 1N).

### 2.2. Hepatic Gene Expression

To investigate the effect of squalene intake on the hepatic transcriptome of pigs with NASH, total RNA was extracted from twelve animals receiving the squalene-enriched steatotic diet and the other twelve receiving the steatotic diet. Four pools made up of three samples for each condition were prepared and analyzed by next generation sequencing using the DNBseq platform. Clean reads (on average 45.8 × 10^6^) were obtained from each library with a coverage of 91.60%. The resultant average mapping ratio with the reference genome (Sscrofa11.1 (GCA_000003025.6)) was 93.51%. On average, 4.33 Gb bases per sample were generated and mapping results for each pool indicated that samples were uniform. A total of 20,430 genes were identified, out of which 20,331 were known and 104 were not previously characterized.

The analysis of transitions showed that A-G were 35,438.5 ± 1884.6 vs. 33,354 ± 1187.5, and C-T were 35,351.8 ± 1844.2 vs. 33,152.3 ± 1089.4 for the control and squalene groups, respectively. Regarding the transversions analyzed, the A-C transversions were 6252 ± 318.6 for the control group and 5886.5 ± 274.0 for the squalene group; A-T transversions were 4462.3 ± 249.5 for the control group and 4111.8 ± 154.7 for the squalene group; C-G transversions were 6728.8 ± 384.0 for the control group and 6297.3 ± 208.2 for the squalene group; and, finally, G-T transversions were 6272.5 ± 312.0 for the control group and 5884.3 ± 260.2 for the squalene group. In quantitative terms, the number of SNPs did not show a significant difference between both groups, thus suggesting that the level of these changes was not influenced by squalene administration.

**Figure 2:**
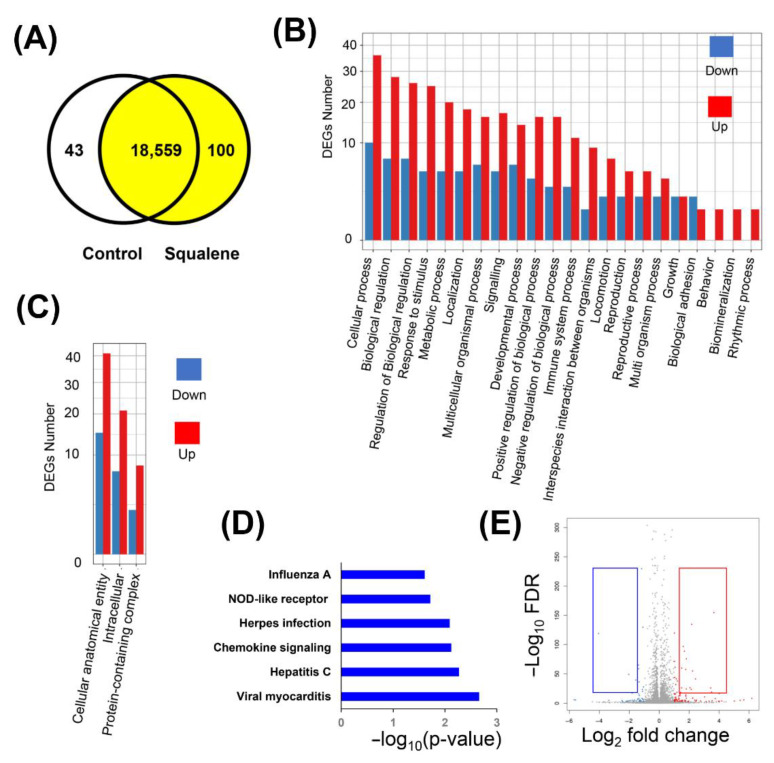
DEGs by the administration of squalene according to RNAseq of livers from a porcine model of dietary NASH development. (**A**) Venn diagram analysis showing the transcripts expressed in control and squalene groups on the steatotic diet with a fold change > 2 and false discovery rate FDR < 0.001. (**B**) Gene ontology (GO) classification of biological processes of up-regulated and down-regulated genes following squalene administration. The *x*-axis illustrates the GO term. The *y*-axis represents the number of up-/down-regulated genes. DEGs, differentially expressed genes. (**C**) GO classification of cellular processes of up-regulated and down-regulated genes by squalene administration. (**D**) Pathway enrichment of DEGs expressed as the log [–P] analyzed by KEGG. (**E**) Volcano plot representing DEGs. Each condition was performed on four pools, each pool consisting of three pig livers. Red and blue squares represent the selected genes shown in Table 1.

Alternative splicing (AS) analyses evidenced five events were significantly modified, namely, 5′ splicing (A5SS), 3′ splicing site (A3SS), retained introns (RI), skipped exons (SE), and mutually exclusive exons (MXE) (data are not shown). Splicing patterns led to differential splicing of genes (DSGs), resulting in a variety of different isoforms from the same gene. Using reference genome annotation, a total of 10,973 novel transcripts were identified. Of these, 9866 represented previously unknown splicing events for known genes, 104 were novel coding transcripts without any known features, and the remaining 1003 were long noncoding RNA. Considering gene expression levels, the control group expressed 18,602 genes compared to 18,659 in the squalene group. There were 100 up-regulated and 40 down-regulated differentially expressed genes (DEGs) in control versus squalene groups (Figure 2A). The gene ontology (GO) classifications of DEGs/up- and down-regulated genes corresponding to biological and cellular processes are displayed in Figure 2B and Figure 2C, respectively. According to the biological classification of GO, the main changes in expression corresponded to 116 genes involved in the cellular process, biological regulation, and its control (Supplementary Table S1). Based on the GO cellular classification, the main changes in expression corresponded to 95 genes involved in cellular anatomical entity, intracellular, and protein-containing complex (Supplementary Table S2). Pathway enrichment of DEGs analyzed according to the Kyoto Encyclopedia of Genes and Genomes (KEGG) (Figure 2D and Supplementary Table S3) showed the main categories corresponding to genes involved in viral and anti-inflammatory responses. The volcano plot of statistical significance against log_2_-fold change between the tested groups is displayed in Figure 2C. Using stringent conditions of signal log_2_ ratio (SL_2_R) above 1.5 or less than −1.5 with a false discovery rate (FDR) of *p* < 0.001, sixteen transcripts were up-regulated, namely, *PPP1R1B*, *OASL*, *PPP4R4*, *HES4*, *NEURL3*, *HTD2*, *CYP2C32*, *TMEM45B*, *AFP*, *ENPEP*, *LOC110256649*, *CYP2J34*, *LOC100526118*, *S100A2*, *SPRY3*, and *FOXG1*, and three were down-regulated, namely, *GTSF1*, *SQLE*, and *CHL1* (Table 1). The biological function of some of these genes could be classified in categories such as metabolism of lipids and xenobiotics, transmembrane transporters, and anti-inflammatory responses.

To confirm sequencing results, all transcripts were individually tested by their corresponding reverse transcriptase quantitative PCR (RT-qPCR) assays (Table 2).

**Figure 3:**
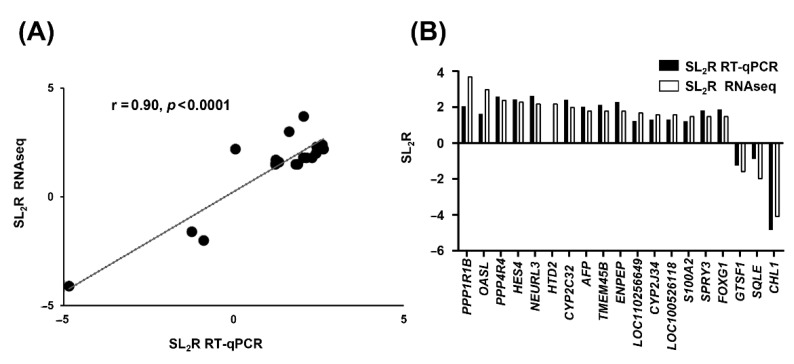
Concordance between used methods of RNA analysis. (**A**) Correlation analysis of 19 selected genes between RNAseq and RT-qPCR normalized to the constitutive *UBA52*. The mean values of SL_2_R of selected genes from RT-qPCR individual analyses (Table 2) were plotted against SL_2_R from RNAseq using pooled samples (Table 1). Twelve samples per group. Excellent agreement between assays was observed (r = 0.90, *p* < 0.0001). (**B**) Difference in expression of the 19 selected genes between both assays.

A correlation study between RNAseq and RT-qPCR methods using the log_2_ fold change of both procedures resulted in an excellent agreement (r = 0.90, *p* < 0.0001) (Figure 3A). Furthermore, all samples were correctly classified (Figure 3B). In pigs receiving squalene (Table 2), twelve transcripts (*PPP1R1B*, *OASL*, *PPP4R4*, *NEURL3*, *TMEM45B*, *AFP*, *ENPEP*, *LOC110256649*, *LOC100526118*, *SPRY3*, *SQLE*, and *CHL1*) underwent significant expression changes.

**Figure 4:**
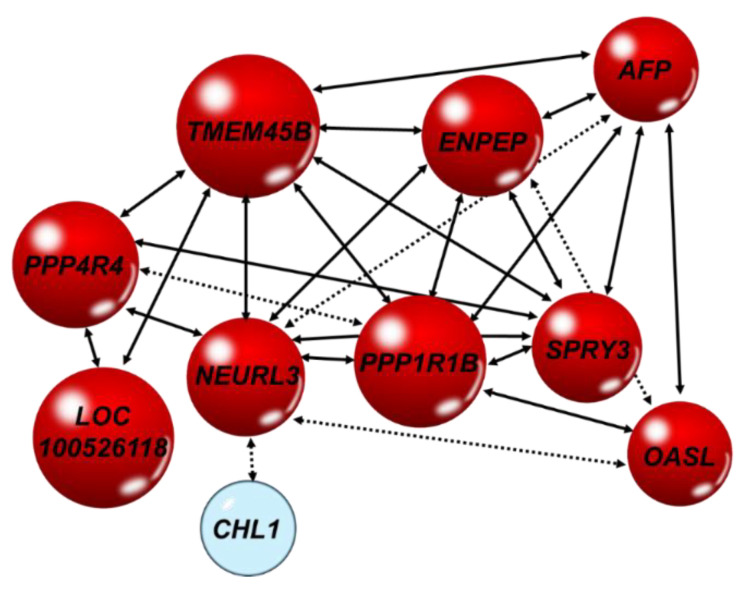
Network of hepatic transcripts in male swine consuming different diets. Significant bilateral Spearman’s correlations of gene expressions analyzed by RT-qPCR. Red, up-regulation and blue, down-regulation. Solid line arrow, *p* < 0.01 and dotted arrow, *p* < 0.02.

Network correlations obtained from RT-qPCR results revealed significant complex relationships among transcripts (Figure 4), particularly for *PPP1R1B*, *OASL*, *PPP4R4*, *NEURL3*, *TMEM45B*, *AFP*, *ENPEP*, and *SPRY3.*

### 2.3. Association among Gene Expression Changes and Pathological Features of NASH

**Figure 5:**
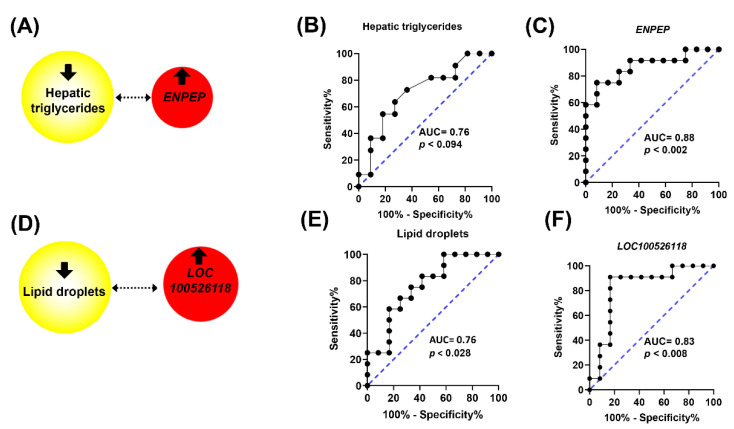
Association among hepatic triglycerides, lipid droplets, and gene expressions in response to squalene administration in pigs. Significant bilateral Spearman’s correlations (*p* < 0.02). Red, up-regulation (**A**,**D**). ROC curves of hepatic triglycerides (**B**), *ENPEP* (**C**), lipid droplets (**E**), and *LOC100526118* (**F**).

**Figure 6:**
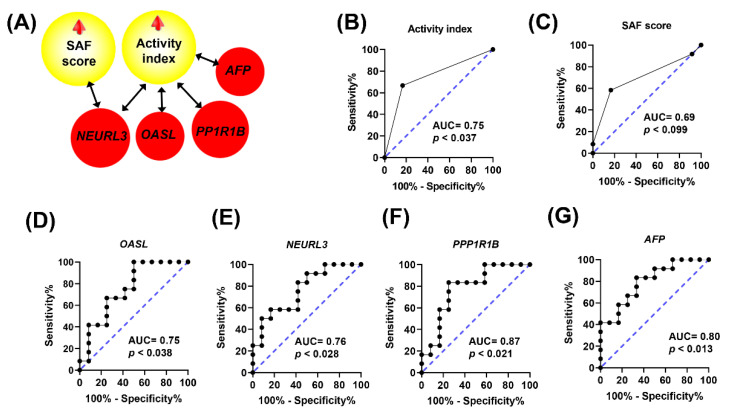
Histological findings and gene expressions in the livers of pigs receiving different diets. Association of NASH parameters with gene expressions (**A**). Significant Spearman’s correlations (*p* < 0.02) are shown. ROC curves of activity index (**B**), SAF score (**C**), hepatic expression of *OASL* (**D**), *NEURL3* (**E**), *PPP1R1B* (**F**), and *AFP* (**G**).

In order to explore the relevance of these gene changes in relation with the hallmarks of NASH, including lipid deposition, ballooning, and inflammation assessed as activity index, and steatosis, activity, and fibrosis as SAF score, correlation and receiver operating characteristic (ROC) curve analyses were carried out. Hepatic triglycerides were negatively associated (ρ = −0.487, *p* < 0.025) with *ENPEP* (Figure 5A) and lipid droplet areas were negatively associated (ρ = −0.473, *p* < 0.026) with *LOC100526118* expression (Figure 5D). Furthermore, changes in *ENPEP* and *LOC100526118* displayed higher discriminant power according to their area under the curve (AUC) values in their ROC curves compared to triglycerides (Figure 5B,C) and lipid droplet areas (Figure 5E,F). The activity index, reflecting the sum of ballooning and inflammation, was found to be positively associated with *AFP*, *NEURL3*, *OASL*, and *PP1R1B* and SAF score was positively associated with *NEURL3* (Figure 6A). Compared to the discriminant power indicated by their AUC values, either activity or SAF scores (Figure 6B,C) were surpassed by the values obtained for *PP1R1B* and *AFP* gene expression changes (Figure 6F,G).

### 2.4. Squalene Accumulates in the Liver and Is Responsible for the Changes in Gene Expressions

**Figure 7:**
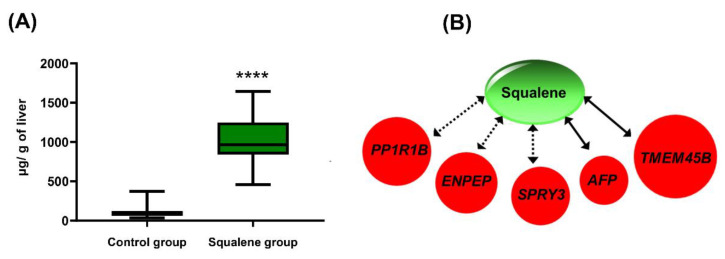
Squalene analyses in male swine consuming different diets. (**A**) Squalene content following dietary regimens. Data are means and 5–95 percentiles for each group of 12 pigs. Statistical analyses were performed according to the one-tail Mann–Whitney U-test. ****, *p* < 0.0001. (**B**) Correlations of hepatic squalene content with gene expression. Red; up-regulation. Significant Spearman’s correlations. Solid line arrows, *p* < 0.01; dotted arrow, *p* < 0.02.

Pigs fed the squalene diet consumed, on average, more solid food than expected, resulting in a squalene intake per animal ranging from 135 to 240 mg/kg per day. The diet enriched with squalene led to a significant accumulation of this triterpene in swine livers (Figure 7A). The squalene content was found to be significantly associated with changes in *PPP1R1B*, *ENPEP*, *SPRY3*, *AFP*, and *TMEM45B* gene expressions, as shown in Figure 7B.

### 2.5. Squalene Is Responsible for the Changes in Genes Expression in Human Hepatoma G2 (HEPG2) and Murine Alpha Mouse Liver 12 (AML12) Cell Lines

To verify whether those changes and associations were a direct response of squalene, the human HEPG2 and the murine AML12 cell lines were incubated in the presence of 30 µM squalene for 72 h. This concentration carried in nanoparticles was particularly effective in controlling reactive oxygen stress in cell cultures [34]. Transcripts displaying significant correlation were assayed. Consistently, in the HEPG2 cell line, *PPP1R1B*, *TMEM45B*, *AFP*, *ENPEP*, and *SPRY3* matched the expression pattern observed in the livers of pigs consuming the squalene-enriched diet (Table 3). The same expression changes were observed in the AML12 cell line, except for *Spry3*, which showed the opposite expression pattern (Table 4).

## 3. Discussion

The nutrigenomic approach aimed to characterize the hepatic transcriptomics of Large White x Landrace pigs developing a reproducible and reversible NASH following a steatotic diet and the changes induced by including 0.5% squalene in the diet. Pigs receiving the latter diet exhibited a noticeable squalene accumulation in their livers (Figure 7A) and significantly increased hepatic expressions of *PPP1R1B*, *OASL*, *PPP4R4*, *NEURL3*, *TMEM45B*, *AFP*, *ENPEP*, *LOC110256649*, *LOC100526118*, and *SPRY3*, as well as significantly decreased expressions of *CHL1* and *SQLE*. All of these transcripts were found to strongly interact in a complex hepatic network of gene expressions (Figure 4). These findings were accompanied by decreased hepatic triglycerides and lipid droplet areas, and an increased cellular ballooning, resulting in a higher NASH activity index and SAF score. However, there were no changes observed in inflammation or fibrosis. The DEGs were notably associated with some of these pathological parameters. In this sense, *ENPEP* was associated with hepatic triglyceride content; *LOC100526118* was associated with lipid droplet areas; *OASL*, *AFP*, *PPP1R1B*, and *NEURL3* were associated with activity index; and *NEURL3* was associated with activity and SAF score. Furthermore, *AFP*, *ENPEP*, and *PPP1R1B* ROC curves showed a great discriminant power according to their areas under the curves. Moreover, the squalene-dependent expression of *PPP1R1B*, *TMEM45B*, *AFP*, *ENPEP*, and *SPRY3* followed the same pattern in vitro using the human HEPG2 hepatic cell line incubated with squalene (Table 3). In murine AML12 cells, squalene induced the same expression profile for selected transcripts, except for *Spry3* (Table 4). These consistent findings suggest a direct effect of squalene on these hepatic transcripts, independent of the studied species. This represents a unique and uncharted territory of gene expression in liver pathology where ballooning is dissociated from steatosis.

The high-throughput RNA-sequencing technology provides raw data about DEGs in addition to polymorphisms, alternative splicing events, and low-expression and novel transcripts [35]. The squalene-modified DEGs were influenced by global alternative splicing events, including 5′ and 3′ sites, retained introns, skipped and mutually exclusive exons, with no change on single nucleotide polymorphisms. RNA pooling assay can be used as an effective tool to reduce the cost of large-scale assays. However, this may lead to random experimental errors in the forms of bias and loss of biological variability [35,36,37]. Therefore, this approach was confirmed by RT-qPCR using individual samples. Nineteen transcripts were analyzed using the stringent criteria of SL_2_R more than 1.5 or less than −1.5 with a false discovery rate (FDR) of *p* < 0.0001. The robust linear association (r = 0.90, *p* < 0.0001) (Figure 3A) and the similitude of response (Figure 3B) verify the concordance between the two technologies and the high reliability of the results.

The steatotic diet used employs high amounts of saturated fat, cholesterol, cholate, and fructose, with low levels of methionine (1.1 g/kg) and choline (50 mg/kg), which are crucial for hepatic β-oxidation and the synthesis of very low-density lipoprotein (VLDL) [33,38]. In this way, the impairment of both metabolic pathways was critical to overcome the natural resistance of pig to develop fatty liver. Moreover, methionine-choline-deficient (MCD) diets lower glycogen stores and induce hepatic oxidative stress, apoptosis, and steatosis [38,39]. In the porcine model used in this study, the administration of the diet over a short period promoted NASH with an identical distribution of cellular ballooning grades 1 and 2, as determined by the steatosis activity fibrosis (SAF) score and FLIP algorithm [32]. The amount of squalene chosen was similar to that administered to rabbits, which induced changes in the hepatic transcriptome [30]. This amount would also represent an adapted metabolic rate of the 1% squalene-containing diets used in mice [29,40].

The porcine model has been used as a human alternative for studying NASH pathogenesis due to their high physiological, anatomical, metabolic, genetic, and liver size similarities [31,41]. The porcine expression map revealed global liver protein-coding and gene expression similarity for both species [42]. Squalene administration to the porcine model reproduced the accumulation seen in the liver of mice [29] and rabbits [25]. However, the latter models showed no change in hepatic triglyceride content despite a higher area of lipid droplets [25], in contrast to the results seen in male pigs. In the pig model, the squalene effect has been tested in animals with established non-alcoholic steatohepatitis. In cultured human hepatocytes, squalene modified the expression of lipid metabolism genes, leading to crucially lower triacylglycerols and cholesterol content, when cellular uptake of fatty acids was increased [43]. In other models, squalene has been found to influence a cluster of genes related to lipid content [29,30]. The difference in responses could be related to species-specific expression patterns or to experimental settings.

To address the meaning of these gene expressions, correlation analyses were conducted, revealing several pathological findings. In this regard, increased *ENPEP* expression was inversely related to the hepatic triglyceride level (Figure 5A). Controversial findings have been described regarding *ENPEP*. It codifies for a membrane glutamyl aminopeptidase whose hepatic levels increase with the progression of NAFLD and decrease upon treatment [44]. However, PPARα modulators that are used as triglyceride-lowering agents induce hepatic expression of *ENPEP* [45,46] and *ENPEP* peptidase protects against hypertriglyceridemia [47]. Interestingly, *ENPEP* was associated with squalene levels and showed the same expression profile in hepatocytes, indicating a direct squalene effect. This was not the case of *LOC100526118*, which codifies for a glutathione S-transferase A1-like that is highly expressed in the liver. This is a highly polymorphic transcript that was activated in response to squalene and was associated with a reduced area of lipid droplets (Figure 5B), probably due to its triglyceride metabolic and antioxidant activities [48,49,50], in addition to detoxification properties due to conjugation of hydrophobic and electrophilic compounds with reduced glutathione (GSH). Decreased GSH has been linked to diminished lipid droplets [51]. *PPP4R4* over-expression was found to activate glucose metabolism [52], leading to lessen triglyceride content [53]. On the other hand, the squalene-reduced expression of *CHL1* impairs insulin secretion [54,55], thus activating the production of hepatic triglyceride-rich lipoproteins [56] and consequent NAFLD [57,58]. Considering the decreases in lipid droplet areas and triglyceride accumulation observed in squalene administration, these gene changes would be offset by the expression of other genes. These findings demonstrate the important involvement of all these genes in hepatic lipid metabolism and their intertwined network to achieve an effect in response to squalene administration.

The squalene accumulation was associated with the ballooning score and, in this way, with NASH activity and SAF score [33]. For this reason, the association of gene expressions with these indexes was examined. As shown in Figure 6, several genes, including *PPP1R1B*, *AFP*, *OASL*, and *NEURL3*, showed a significant association with those parameters. All these transcripts were noticeably interacting in a complex network, thus suggesting a functional association (Figure 4). *PPP1R1B* suppresses protein phosphatase 1 [59] and induces glycogenosis [60]. Glycogenosis in NAFLD patients is associated with higher ballooning score, despite the lower steatosis and fibrosis scores [61]. The same profile was observed in NASH liver in response to squalene. Furthermore, *AFP* is a fatty acid binding protein. Fatty acid composition triggers different responses to NAFLD and its synthesis is correlated with ballooning score [62]. In the liver, a lower estearic/palmitic fatty acid ratio and a higher palmitoleic/palmitic ratio increase ballooning and fibrosis scores [62], while the serum level of palmitoleic fatty acid is negatively correlated with NAFLD activity and ballooning scores [63]. Interestingly, in the squalene-NASH swine model, the plasma lipidomic showed a consistent decrease in palmitic fatty acid [33]. Moreover, *AFP* is essential in the SMADS signal transduction pathway against hepatic fibrosis, particularly for SMAD2 [64,65]. Both *PPP1R1B* and *AFP* showed the same expression profile in vitro in hepatocytes, indicating a direct squalene effect. Furthermore, *OASL* represses interferon type 1 activity [66], therefore reducing the polarization of macrophages, and consequently suppressing inflammation of NAFLD [67], reinforcing the anti-inflammatory role of intervention. Notably, *OASL* is positively regulated by PPARα, which is inversely related to NAFLD activity score [68]. Finally, *NEURL3* enables ubiquitin protein ligase activity 3, which is involved in down-regulating NAFLD gene clusters [69,70], thus alleviating the progress of NAFLD. The combined induced expression of *PPP1R1B*, *AFP*, *OASL*, and *NEURL3* may play an important role in the development of this ailment. In fact, the obtained ROC curves suggest that *AFP* and *PPP1R1B* have a stronger discriminating value than classical indexes of NAFLD activity and SAF score. In humans, *PPP1R1B* and *AFP* are positively associated with ballooning score [60,61,62]. In this way, this model reproduces the human situation, but the expression of *ENPEP*, which lowers triglyceride levels [45,46,47], and that of *NEURL3* and *OASL*, which suppress inflammatory response [66,67], would create a unique setting of ballooning and no inflammatory or fibrotic changes, which merit further research.

Previous studies showed that squalene exerts anti-neoplastic effects [30,71]. *PPP1R1B* is activated in colorectal liver metastasis [72]. *SPRY3* is a negative regulator of the RTK/Ras/MAPKS pathway. However, unlike *SPRY1*, *SPRY2*, and *SPRY4*, and despite its positive linkage with *SPRY1*, the expression level in HCC is not modified [73]. *TMEM45B* is a hub gene in hepatic differentiation [74] and is up-regulated in response to cirrhosis [75]. In addition, it has distinct neoplastic expression patterns, although none are of hepatic origin [76,77,78,79], so further studies are required to demonstrate the effect of *TMEM45B* on hepatic neoplasia. Both *SPRY3* and *TMEM45B* were induced by squalene administration (Table 2). On the other hand, *CHL1* is a modulator of the cell cycle through the p53 pathway, with frequent overexpression in liver cancers [80]. In this case, squalene administration decreased its expression, a phenomenon that seems to be independent of the previous expression since they were not connected in the proposed gene network (Figure 4). Therefore, the phenotype could be a balance of expression among these genes, which could play a role in the progression of NASH to cirrhosis and cancer.

In conclusion, feeding male Large White x Landrace pigs a squalene-supplemented steatotic diet resulted in squalene accumulation in the liver and acted as a modulator of the hepatic transcriptome. Transcripts including *PPP1R1B*, *OASL*, *PPP4R4*, *NEURL3*, *AFP*, *TMEM45B*, *ENPEP*, *LOC110256649*, *LOC100526118*, *SPRY3*, *CHL1*, and *SQLE* experienced significant changes, and their expression was significantly associated, suggesting an interacting network. Concomitantly, these findings were accompanied by decreased hepatic levels of triglycerides and lipid droplets, whereas cellular ballooning score, NASH activity, and SAF score increased. The associations of gene expression with pathological features indicate that *LOC100526118* was associated with hepatic lipid droplet areas; *ENPEP* with hepatic triglycerides; *OASL*, *AFP*, and *PPP1R1B* with NASH activity; and *NEURL3* with NASH activity and SAF scores. Moreover, *AFP*, *ENPEP*, *LOC100526118*, and *PPP1R1B* showed a strong discriminant power compared to those pathological parameters. Several transcripts, namely, *PPP1R1B*, *TMEM45B*, *AFP*, and *ENPEP*, showed the same expression pattern in vitro in a human cell line, indicating a hepatic direct relationship between squalene and these gene expressions. Overall, these findings indicate that squalene accumulates in the liver and is able to modulate gene expression changes that may influence the fate of NASH.

## 4. Materials and Methods

### 4.1. Animal Models and Experimental Design

The experimental animals were 24 male Large White x Landrace pigs generated by artificial insemination at Cooperativa Ganadera de Caspe (Zaragoza, Spain). After one month of acclimation in the facilities of Servicio General de Apoyo a la Investigación, División de Experimentación Animal, Facultad de Veterinaria, Universidad de Zaragoza, pigs, weighing 38 ± 2.8 kg, received a steatotic diet of high cholate, cholesterol, fructose, and saturated fat, and low methionine and choline content, for 1 month [32]. Then, they were liver biopsied and divided into two groups of equal hepatic triglyceride and cholesterol contents. The first group (*n* = 12) was fed with the steatotic diet and the second (*n* = 12) was fed with the same diet enriched with 0.5% squalene. Both groups received their diets for one additional month. Dietary consumption was monitored on a weekly basis. Taking into account the amount consumed and body weight, the dose was equivalent to 135 mg/kg dose of squalene per animal per day. Squalene was purchased from Molekula Group (Darlington, UK). Animals had ad libitum access to feed and water. Following the experimental period and after an overnight fast, all pigs were euthanized by an overdose of Propofol (B/Braun-Vetcare, Rubí, Barcelona, Spain) and biological specimens were collected. Experiments were carried out according to the European Union guidelines for laboratory animals (Directive 2010/63/UE) and in compliance with ARRIVE guidelines. Study protocols were authorized by the Ethics Committee for Animal Research of the University of Zaragoza (PI43/15).

### 4.2. Liver Histological Analyses

A sample from each liver was stored in neutral formaldehyde and embedded in paraffin wax. Sections (4 μm) were stained with hematoxylin-eosin or with Masson’s trichrome staining and then images were captured using a Nikon microscope. Lipid droplet and fiber areas were quantified and expressed as percentage of total liver section with Adobe Photoshop CS3 (Adobe Inc., San Jose, CA, USA). Liver sections were blindly assessed by a single qualified pathologist. The fatty liver inhibition of progression (FLIP) algorithm and SAF score were used to categorize the histological stages of NASH [5]. Briefly, steatosis was based on the percentage of hepatocytes that contained large and medium lipid droplets, but no microvesicles, using a scale of 0 to 3 (0: <5%; 1: 5–33%, 2: 34–66% and 3: >67%). Ballooning was graded on a scale from 0 to 2 (0: normal hepatocytes; 1: groups of rounded hepatocytes with pale cytoplasm and the size similar to that of normal hepatocytes; and 2: as for grade 1, but where there was at least one enlarged ballooned hepatocyte (2-fold size compared with that of normal cells within a cluster of grade 1 hepatocytes)). Inflammation was assessed by counting the number of inflammatory foci ((2 or more cells)/lobule) at 20× magnification. The inflammation was graded 0 for none, 1 for less than 2 foci per lobule, and 2 for at least 2 foci per lobule. The activity score was calculated by adding the grades of ballooning and lobular inflammation. Fibrosis was classified on a scale from 0 to 4 based on the location and number of fibers present. Grade 0 indicated no fibrosis present, *g*rade 1 indicated the presence of fibrosis limited to the perisinusoidal or periportal regions, *g*rade 2 indicated the presence of perisinusoidal and periportal fibrosis without flanges, *g*rade 3 indicated the presence of flanged fibrosis, and *g*rade 4 indicated the presence of cirrhosis. The steatosis activity fibrosis (SAF) score was calculated by adding together the scores for steatosis, activity, and fibrosis.

### 4.3. Quantification of Hepatic Lipids and Squalene

Hepatic cholesterol and triglyceride extraction and analyses were carried out as previously described [32]. Squalene was extracted and quantified by solid phase extraction, gas chromatography, and mass spectrometry (GC/MS), as mentioned [25].

### 4.4. RNA Extraction

Total liver RNA was extracted using the Quick-RNA MiniPrep kit (Cat. No: R1055, ZYMO Research, Irvine, CA, USA), following the manufacturer’s instructions. Both RNA quantity and purity were assessed using a Nanodrop1000 Spectrophotometer (Thermo Scientific, Waltham, MA, USA). RNA was quantified by measuring the absorbance at 260 nm. Purity was determined by analysis of the absorbance at A260/A280. The ratio was ~2. The integrity for both 28S and 18S ribosomal RNAs was verified by agarose gel electrophoresis with the 28S/18S ratio being greater than 2. The quality of RNAseq samples (RQI > 9) was checked using Bio-Rad Lab Chip technology (Hercules, CA, USA).

### 4.5. RNAseq and Data Analyses

For both animal groups, four pools of three animals each were prepared using equal amounts of hepatic total RNA. RNA quantity and quality were tested by RIN value, 28S/18S, and fragment length distribution using an Agilent 2100 Bio analyzer (Agilent RNA 6000 Nano Kit, Santa Clara, CA, USA). Library construction and sequencing were carried out at the BGI service (Shenzhen, China) as previously described [81]. Sequencing reads containing low-quality, adaptor-polluted, and unknown base reads were filtered to obtain clean reads. These reads were mapped onto the reference genome (Sscrofa11.1 (GCA_000003025.6)) using HISAT2. The detailed bioinformatics workflow was previously described [32]. The complete database was deposited in the GEO database with the accession number GSE214732.

### 4.6. Reverse Transcriptase-Quantitative PCR (RT-qPCR)

RNAseq transcripts displaying a signal log_2_ ratio > 1.5 or <−1.5 and FDRs lower than 0.001 were selected for confirmation in individual samples by RT-qPCR. Primers were designed using NCBI and then checked for gene specificity and full variant coverage by BLAST (NCBI), KEGG and Ensemble Genome Browser (Supplementary Table S4). Equal amounts (500 ng) of DNA-free RNA were used to synthesize complementary DNA (cDNA) using PrimeScript RT reagent Kit (Cat. No: RR037A, Takara, Kutsatsu, Shiga, Japan) following the manufacturer’s instructions. Primer concentrations and cDNA input were optimized to obtain efficiencies between 95 and 105%. Quantitative real-time analysis was carried out using SYBR Green dye master mix (Applied Biosystems, Foster city, CA, USA) according to the manufacturer’s instructions, utilizing a ViiA7 Real-TIME PCR System (Life Technologies, Carlsbad, CA, USA). Analysis of relative gene expression data was conducted using the 2^−ΔΔCT^ method and normalized to the most stable reference genes: UBA52 for swine samples, *GAPDH* for the HEPG2 cell line, and *Ppib* for the AML12 cell line.

### 4.7. AML12 Cell Culture

The murine hepatic cell line was obtained from the ATCC collection (Manassas, VA, USA) and was grown in a humidified atmosphere of 5% CO_2_ at 37 °C in Dulbecco’s modified Eagle’s minimum essential medium (DMEM) with glucose (4.5 g/L) (Thermo Fisher Scientific Waltham, MA, USA), F12-Ham’s medium with 1 mM l-glutamine (GE Healthcare Life Science, South Logan, UT, USA) enriched with 10% fetal bovine serum (Thermo Fisher Scientific Waltham, MA, USA), 1:500 insulin-transferrin-selenium (Corning, Bedford, MA, USA), 40 ng/mL dexamethasone (Sigma-Aldrich; Merck Millipore, Darmstadt, Germany), 1% non-essential amino acids (Thermo Fisher Scientific, Waltham, MA, USA), 100 U/mL penicillin; Thermo Fisher Scientific), 100 µg/mL streptomycin; Thermo Fisher Scientific, Waltham, MA, USA), and 2.5 µg/mL amphotericin B (Thermo Fisher Scientific, Waltham, MA, USA). When cells reached a confluence of 90–100%, the medium was removed and cells were washed twice with phosphate buffered saline followed by addition of medium free of fetal bovine serum and amphotericin B. Cells were then incubated for 72 h with 30 µM squalene (Sigma-Merck, Darmstadt, Germany), carried in 0.1% poly lactic-co-glycolic acid (PLGA) versus non-loaded PLGA nanoparticles as control [34]. Each condition was tested in triplicate in two experiments. Media were removed, and cells were washed twice with phosphate buffered saline and collected. Squalene effect was investigated at mRNA level for genes (*Ppp1r1b*, *Enpep*, *Afp*, *Tmem45b*, and *Spry3*), showing a significant association with hepatic squalene content in the swine model. The primers are listed in Supplementary Table S4.

### 4.8. HEPG2 Cell Culture

The human hepatic cell line was obtained from the ATCC collection and was grown in a humidified atmosphere of 5% CO_2_ at 37 °C in Dulbecco’s modified Eagle’s minimum essential medium (DMEM) (4.5 g/L) (Thermo Fisher Scientific Waltham, MA, USA) supplemented with 10% fetal bovine serum (Thermo Fisher Scientific, Waltham, MA, USA), 2% of 4 mM glutamine, 1% of 100 mM sodium pyruvate, 1% non-essential amino acids (Thermo Fisher Scientific, Waltham, MA, USA), 100 U/mL penicillin; Thermo Fisher Scientific), 100 µg/mL streptomycin; Thermo Fisher Scientific, Waltham, MA, USA), and 2.5 µg/mL amphotericin B (Thermo Fisher Scientific, Waltham, MA, USA). When cells reached 90–100% confluence, the medium was removed and cells were washed twice with phosphate buffered saline followed by addition of the medium free of fetal bovine serum and amphotericin B. Cells were then incubated for 72 h with 30 µM squalene (Sigma-Merck, Darmstadt, Germany), carried in 0.1% poly lactic-co-glycolic acid (PLGA) versus non-loaded PLGA nanoparticles as control [34]. Each condition was tested in triplicate. Media were removed, and cells were washed twice with phosphate buffered saline and collected. Squalene effect was investigated at mRNA level for genes (*PPP1R1B*, *ENPEP*, *AFP*, *TMEM45B*, and *SPRY3*). Primers are listed in Supplementary Table S4.

### 4.9. Quality Control and Statistics

Samples in quantitative real-time analysis were run in duplicate and their coefficient of variation was obtained. Duplicates showing a coefficient of variation greater than 3% were discarded and repeated. Statistical analyses were carried out with GraphPad Prism 8.0 for Windows (GraphPad Software, San Diego, CA, USA). Data were analyzed for normal distribution using the Shapiro–Wilk test and for homogeneity of variance using Bartlett’s F-test. In most cases, the outcome of these parameters failed, and results were analyzed by the nonparametric one-tailed Mann–Whitney U-test. *p* < 0.05 was considered significant. Receiver operating characteristic (ROC) curves were generated using GraphPad Prism 8.0 for quantitative values. This software also reports the area under the curve (AUC), which defines how well the measured parameter can differentiate between tested groups for each parameter. Correlations among all parameters were analyzed using two-tailed Spearman’s correlation coefficient according to the Statistical Package for Social Sciences version 25 (IBM, Armonk, NY, USA), and those with *p* < 0.02 were considered.

## Figures and Tables

**Table 1:** The most striking hepatic transcripts regulated by 0.5% squalene in male pigs consuming the steatotic diet according to RNAseq.

Function	Gene ID	Name	Symbol	Control (FPKM)	Squalene (FPKM)	Log_2_ Fold Change Squalene/Control
**Up-regulated**						
Signal transduction	100736966	Protein phosphatase 1 regulatory inhibitor subunit 1B	*PPP1R1B*	63	785	3.7
Signal transduction	595119	2′-5′-oligoadenylate synthase-like protein	*OASL*	441	3611	3.0
Signal transduction	100737576	Protein phosphatase 4 regulatory subunit 4	*PPP4R4*	59	314	2.4
Transcription factor	100739264	Hes family bHLH transcription factor 4	*HES4*	569	2853	2.3
Protein ubiquinization	100524797	Neuralized E3 ubiquitin protein ligase 3	*NEURL3*	98	456	2.2
Fatty acid biosynthesis	100515579	Hydroxyacyl-thioester dehydratase type 2, mitochondrial	*LOC100515579* (*HTD2*)	259	1155	2.2
Metabolism of xenobiotics	403106	Cytochrome P450, 2C32	*CYP2C32*	2165	8633	2.0
Membrane protein	100516991	Transmembrane protein 45B	*TMEM45B*	237	825	1.8
Fatty acid transport	397586	Alpha-fetoprotein	*AFP*	77	268	1.8
Peptide hormone metabolism	397080	Glutamyl aminopeptidase	*ENPEP*	191	647	1.8
Endogenous retrovirus C	110256649	Porcine endogenous retrovirus C gag protein	*LOC110256649*	337	1071	1.7
Metabolism of xenobiotics	100524750	Cytochrome P450, 2J34	*CYP2J34*	414	1249	1.6
Metabolism of xenobiotics	100526118	Glutathione S-transferase A1-like	*LOC100526118*	5125	15029	1.6
Cell cycle progression	100152729	S100 calcium binding protein A2	*S100A2*	1817	5171	1.5
ERK signaling	100515521	Sprouty RTK signaling antagonist 3	*SPRY3*	301	848	1.5
Transcription factor	110261579	Forkhead box G1	*FOXG1*	134	376	1.5
**Down-regulated**						
Control of transcription	100521495	Gametocyte specific factor 1	*GTSF1*	543	182	−1.6
Sterol biosynthesis	100113409	Squalene monooxygenase	*SQLE*	464	112	−2.0
Signal transduction	100511780	Cell adhesion molecule L1 like	*CHL1*	570	34	−4.1

Data are fragments per kilo base per million mapped (FPKM) reads. Only genes with SL_2_R higher than 1.5 or lower than −1.5, FDR < 0.0001, and counts in more than 75% of samples have been taken into consideration. Annotations were carried out using https://www.ensembl.org/Pig/Search/Results?q=;site=ensembl;facet_species=Pig (accessed on 30 May 2023), https://www.rnaatlas.org/ (accessed on 30 May 2023), and https://blast.ncbi.nlm.nih.gov/Blast.cgi (accessed on 30 May 2023) against Sus scrofa 11.1 reference Annotation Release 106 Biological function obtained from https://www.genecards.org/ (accessed on 30 May 2023) and https://www.uniprot.org/uniprot/ (accessed on 30 May 2023).

**Table 2:** Changes in selected hepatic gene expressions in response to 0.5% squalene in male pigs consuming the steatotic diet according to RT-qPCR assay.

Gene Symbol	Control (*n* = 12)	Squalene (*n* = 12)	Fold Change	Signal log_2_ Ratio (SL_2_R)
*PPP1R1B*	2.2 ± 3.1	9.0 ± 10.5 *	4.2	2.1
*OASL*	0.8 ± 0.7	2.5 ± 1.4 *	3.1	1.6
*PPP4R4*	1.1 ± 0.8	6.7 ± 13.2 *	6.1	2.6
*HES4*	1.3 ± 1.3	7.2 ± 20.2	*5.4*	*2.4*
*NEURL3*	1.8 ± 2.2	11.3 ± 23.6 *	6.2	2.6
*LOC100515579* (*HTD2*)	1.1 ± 0.4	1.1 ± 0.8	1.0	0.05
*CYP2C32*	2.8 ± 4.7	15.2 ± 24.6	5.3	2.4
*TMEM45B*	2.5 ± 4.7	11.1 ± 15.8 *	4.4	2.1
*AFP*	1.3 ± 0.9	5.4 ± 8.5 **	4.1	2.0
*ENPEP*	1.3 ± 0.7	6.3 ± 9.7 **	4.9	2.3
*LOC110256649*	1.6 ± 1.7	3.7 ± 3.2 *	2.4	1.2
*CYP2J34*	2.0 ± 1.6	4.9 ± 9.6	2.5	1.3
*LOC100526118*	1.4 ± 1.6	3.5 ± 4.8 **	2.5	1.3
*S100A2*	1.9 ± 1.9	4.4 ± 7.0	2.3	1.2
*SPRY3*	1.2 ± 0.7	4.3 ± 5.2 *	3.5	1.8
*FOXG1*	1.9 ± 2.2	6.8 ± 11.7	3.7	1.9
*GTSF1*	4.0 ± 5.7	1.7 ± 1.7	0.4	−1.2
*SQLE*	5.1 ± 14.5	2.7 ± 1.9 *	0.5	−0.9
*CHL1*	6.5 ± 11.9	0.2 ± 0.2 *	0.03	−4.8

Results are expressed as means and standard deviations normalized to *UBA52*. Twelve samples per group. Statistical analysis was carried out according to the Mann–Whitney U-test; *, *p* < 0.05 and **, *p* < 0.01.

**Table 3:** Changes in selected hepatic gene expressions in response to 30 µM squalene in HEPG2 cell line according to RT-qPCR assay.

Gene Symbol	Control (*n* = 6)	Squalene (*n* = 6)	Fold Change	Signal log_2_ Ratio
*PPP1R1B*	0.7 ± 0.2	0.9 ± 0.2 *	1.3	0.4
*TMEM45B*	1.0 ± 0.1	1.3 ± 0.1 **	1.3	0.4
*AFP*	0.8 ± 0.2	1.0 ± 0.1 *	1.2	0.2
*ENPEP*	0.8 ± 0.2	1.5 ± 0.3 *	1.9	0.9
*SPRY3*	0.8 ± 0.3	1.4 ± 0.3 *	1.8	0.8

Results are expressed as means and standard deviations normalized to GAPDH. Six replicates per group. Statistical analysis was carried out according to Mann–Whitney U-test. *, *p* < 0.05 and **, *p* < 0.01.

**Table 4:** Changes in selected hepatic gene expressions in response to 30 µM squalene in AML12 cell line according to RT-qPCR assay.

Gene Symbol	Control (*n* = 6)	Squalene (*n* = 6)	Fold Change	Signal log_2_ Ratio
*Ppp1r1b*	1.0 ± 0.1	1.5 ± 0.5 *	1.5	0.6
*Tmem45b*	1.0 ± 0.3	3.0 ± 1.7 *	3.0	1.6
*Afp*	1.0 ± 0.1	1.6 ± 0.8 *	1.6	0.7
*Enpep*	1.0 ± 0.1	1.4 ± 0.5 *	1.4	0.5
*Spry3*	1.2 ± 0.1	0.7 ± 0.1 *	0.7	−0.6

Results are expressed as means and standard deviations normalized to Ppib. Six replicates each group. Statistical analysis was carried out according to Mann–Whitney U-test; *, *p* < 0.05.

## Data Availability

RNAseq data are available at GEO database with the accession number GSE214732. The rest will be provided on a reasonable request.

## Supplementary Materials

The supporting information can be downloaded at: https://www.mdpi.com/article/10.3390/ijms241612552/s1.
