# Supplementary material for: Differentially Expressed Genes in Response to a Squalene-Supplemented Diet Are Accurate Discriminants of Porcine Non-Alcoholic Steatohepatitis
Source: Int J Mol Sci. 2023 Aug 8;24(16):12552. doi: 10.3390/ijms241612552 (PMC10454218; doi:10.3390/ijms241612552)
Supplement: Supplementary file 1 [file ijms-24-12552-s001.zip › ijms-2486318-supplementary.pdf]

Supplementary Table S1. Genes comprised in the main categories displayed in Figure 2B of gene ontology classification of biological processes of up-regulated and down-regulated genes.

**GO term**	**Regulation**	**Number of annotated genes**	**Gene symbols**
Cellular process	Up	36	*AFP, ANO3, BORCS6, CBLC, CCL20, CDK1, CDK3, CLDN6, CXCL10, CXCL8, ENPEP, FOXG1, HTRA3, IFIT2, IFIT3, INHBB, ISG15, KCTD14, LIF, LOC100157935, LOC100516302, LOC110255217, MLC1, PKP3, PLP1, PLPPR2, PPP1R1B, RGS5, RSAD2, SDCBP2, SEMA3A, SERPINB2, SLITRK5, SPRY3, SYCE1L, TMIGD3*
	Down	10	*APOD, CA3, CRABP1, DEFB1, KCNJ3, LOC100621701, NPSR1, PIWIL2, RASL10A, SYT17*
Biological regulation	Up	28	*AFP, ANO3, CBLC, CCL20, CDK1, CDK3, CXCL10, CXCL8, ENPEP, FOXG1, HTRA3, IFIT3, INHBB, ISG15, LIF, LOC100516302, LOC110255217, MLC1, PKP3, PLP1, PLPPR2, RGS5, RSAD2, SEMA3A, SERPINB2, SLITRK5, SPRY3, TMIGD3*
	Down	7	*APOD, CRABP1, DEFB1, NPSR1, PIWIL2, RASL10A, SYT17*
Regulation of biological process	Up	26	*AFP, CBLC, CCL20, CDK1, CDK3, CXCL10, CXCL8, FOXG1, HTRA3, IFIT3, INHBB, ISG15, LIF, LOC100516302, LOC110255217, MLC1, PKP3, PLP1, PLPPR2, RGS5, RSAD2, SEMA3A, SERPINB2, SLITRK5, SPRY3, TMIGD3*
	Down	7	*APOD, CRABP1, DEFB1, NPSR1, PIWIL2, RASL10A, SYT17*
Response to stimulus	Up	25	*AFP, CBLC, CCL20, CDK1, CDK3, CXCL10, CXCL8, FOXG1, IFIT2, IFIT3, INHBB, ISG15, LIF, LOC100153139, LOC106504372, LOC110255217, MLC1, OASL, PLP1, PLPPR2, RGS5, RSAD2, SEMA3A, SLITRK5, SPRY3*
	Down	5	*APOD, CRABP1, DEFB1, LOC100525396, RASL10A*
Metabolic process	Up	20	*AFP, CBLC, CCL20, CDK1, CDK3, CXCL8, ENPEP, FOXG1, INHBB, ISG15, LIF, LOC100157935, LOC100516302, LOC100526118, MMP1, PKP3, PLP1, PLPPR2, RSAD2, TMIGD3*
	Down	5	*APOD, CA3, CRABP1, PIWIL2, SQLE*
Localization	Up	18	*AFP, ANO3, BORCS6, CCL20, CDK1, CXCL10, CXCL8, ENPEP, FOXG1, INHBB, LIF, LOC100157935, MLC1, PKP3, RSAD2, SEMA3A, SYCE1L, TMIGD3*
	Down	5	*APOD, DEFB1, KCNJ3, NPSR1, SYT17*
Multicellular organismal process	Up	16	*AFP, CDK1, CDK3, CXCL10, CXCL8, ENPEP, FOXG1, GUCA1A, INHBB, ISG15,*

			LIF, PLP1, RSAD2, SEMA3A, SLITRK5, SPRY3
	Down	6	*APOD, CRABP1, DEFB1, LOC100621701, PIWIL2, SYT17*
Signaling	Up	17	*AFP, CBLC, CCL20, CDK3, CXCL10, CXCL8, INHBB, LIF, LOC110255217, PLP1, PLPPR2, PPP1R1B, RGS5, RSAD2, SEMA3A, SLITRK5, SPRY3*
	Down	5	*APOD, CRABP1, DEFB1, LOC100621701, RASL10A*
Developmental process	Up	14	*AFP, CDK1, CDK3, CXCL10, CXCL8, FOXG1, INHBB, ISG15, LIF, PLP1, RSAD2, SEMA3A, SLITRK5, SPRY3*
	Down	6	*APOD, CRABP1, DEFB1, LOC100621701, PIWIL2, SYT17*
Positive regulation of biological process	Up	16	*CCL20, CDK1, CDK3, CXCL10, CXCL8, FOXG1, INHBB, ISG15, LIF, LOC110255217, MLC1, PKP3, PLP1, RSAD2, SEMA3A, SLITRK5*
	Down	4	*DEFB1, NPSR1, PIWIL2, SYT17*
Negative regulation of biological process	Up	16	*CBLC, CDK1, CXCL10, CXCL8, FOXG1, IFIT3, INHBB, ISG15, LIF, LOC100516302, PKP3, RGS5, RSAD2, SEMA3A, SERPINB2, TMIGD3*
	Down	3	*APOD, NPSR1, PIWIL2,*
Immune system process	Up	11	*CCL20, CXCL10, CXCL8, IFIT3, ISG15, LIF, LOC100153139, LOC106504372, LOC110255217, OASL, RSAD2*
	Down	3	*APOD, DEFB1, LOC100525396*
Interspecies interaction among organisms	Up	9	*CCL20, CXCL10, CXCL8, IFIT3, ISG15, LOC110255217, OASL, RSAD2, SYCE1L*
	Down	1	*DEFB1*
Locomotion	Up	7	*CCL20, CXCL8, CXCL10, ENPEP, FOXG1, SEMA3A, TMIGD3*
	Down	2	*APOD, DEFB1*
Reproduction	Up	5	*AFP, CDK1, INHBB, LIF, SEMA3A*
	Down	2	*DEFB1, PIWIL2*
Reproductive process	Up	5	*AFP, CDK1, INHBB, LIF, SEMA3A*
	Down	2	*DEFB1, PIWIL2*
Multi-organism process	Up	4	*AFP, CDK1, INHBB, LIF*
	Down	2	*DEFB1, PIWIL2*
Growth	Up	2	*HTRA3, SEMA3A*
	Down	2	*APOD, SYT17*
Biological adhesion	Up	1	*CXCL8*
	Down	2	*APOD, LOC100621701*
Behavior	Up	1	*SLITRK5*
	Down	-	-
Biomineralization	Up	1	*ISG15*
	Down	-	-
Rhythmic process	Up	1	*AFP*
	Down	-	-

Supplementary Table S2. Genes comprised in the main categories displayed in Figure 2C of gene ontology classification of cellular processes of genes.

GO term	Regulation	Number of annotated genes	Gene symbols
Cellular anatomical entity	Up	41	*AFP, ANO3, BORCS6, CBLC, CCL20, CDK1, CDK3, CLDN6, CXCL10, CXCL8, ENPEP, FOXG1, GUCA1A, HTRA3, IFIT3, INHBB, ISG15, LIF, LOC100153139, LOC100157935, LOC100516302, LOC106504372, LOC110255217, MLC1, MMP1, NRM, PI15, PKP3, PLP1, PLPPR2, RGS5, RSAD2, SDCBP2, SEMA3A, SERPINB2, SERPINB7, SLITRK5, SPRY3, SYCE1L, TMEM45B, TMIGD3*
	Down	15	*APOD, CA3, CPNE4, CRABP1, DEFB1, KCNJ3, LOC100525396, LOC100621701, NPSR1, PIWIL2, RASL10A, SHISA3, SQLE, SYT17, TECTB*
Intracellular	Up	21	*AFP, BORCS6, CBLC, CCL20, CDK1, CDK3, CXCL8, ENPEP, FOXG1, IFIT3, INHBB, LIF, LOC100157935, LOC110255217, MLC1, NRM, PKP3, RGS5, RSAD2, SDCBP2, SYCE1L*
	Down	7	*APOD, CA3, CRABP1, DEFB1, PIWIL2, SQLE, SYT17*
Protein-containing complex	Up	8	*BORCS6, CDK1, CDK3, LOC100153139, LOC100157935, LOC106504372, PKP3, SLITRK5*
	Down	3	*APOD, KCNJ3, PIWIL2*

Supplementary Table S3. Genes comprised in the main categories displayed in Figure 2D according to Kyoto Encyclopedia of Genes and Genomes (KEGG).

**Pathways**	**Number of annotated genes**	**Differentially expressed genes**
Viral myocarditis	5	*LOC110256649, LOC106504372, LOC100153139),* *LOC110258821* and *BGI_novel_G000045*
Hepatitis C	5	*IFIT1, IFIT3, IFIT2, CXCL8* and *CLDN6*
Chemokine signaling	6	*CXCL10, BGI_novel_G000048, LOC100525396, CCL20, CXCL8* and *LOC110255211*
Herpes infection	8	*IFIT1, IFIT3, IFIT2, LOC106504372, LOC100153139, CDK1, MAP3K7CL* and *SHISA3*
NOD-like receptor	5	*GVIN1, BGI_novel_G000048, LOC100525396, CXCL8* and *MAP3K7CL*
Influenza A	5	*CXCL10, RSAD2, LOC106504372, LOC100153139* and *CXCL8*

Supplementary Table S4. Characteristics of primers used in RT-qPCR according to MIQE guidelines.

Gene symbol	Accession		Sequence	Amplicon length	Exons	[Primer]	Efficiency
Sus scrofa							
PPP1R1B	XM_021067044.1<br>XM_021067045.1<br>XM_021067046.1<br>XM_021067047.1	Sense<br>Antisense	GGCACCACCTCAAGTCGAAG<br>TGATGGACTGCAGGTGAGAC	99	NA	0.08 µM	99%
OASL	NM_001031790.1	Sense<br>Antisense	GTGTGGTTGGTCGAGGAAGT<br>CTGTAGGCAGGCATGATGGT	98	2	0.08 µM	101%
PPP4R4	XM_021099655.1<br>XM_021099656.1<br>XM_021099657.1<br>XM_021099658.1<br>XM_021099659.1<br>XM_021099660.1<br>XM_021099661.1<br>XM_021099662.1	Sense<br>Antisense	ATATGCCTGTCTCCCACACG<br>TGAAGCTGCCTTTTGCACAG	115	NA	0.2 µM	97 %
HES4	XM_003481929.4	Sense<br>Antisense	GAAAGAGAGTTCCCGCCACT<br>GACCAGGAATCGGTTCACCT	181	NA	0.2 µM	99 %
NEURL3	XM_003124839.5	Sense<br>Antisense	CATGGATCCTACAGCCAGCG<br>GGCATTTGGCCGTGTCTTTG	203	NA	0.2 µM	102%
HTD2	XM_003132274.4<br>XM_021069097.1<br>XM_021069098.1<br>XM_003132275.4	Sense<br>Antisense	GTGGGGTTGGACTGAATGCT<br>ATGTCCAAGGGTAAGGCAGC	97	NA	0.2 µM	98%
CYP2C32	XM_013983597.2	Sense<br>Antisense	GCCCGCATGGAGCTATTTTT<br>CGTGGCTGAACCCACTATAAA	115	NA	0.08 µM	96%
AFP	NM_214317.1	Sense<br>Antisense	AGAGGAACAACTTGAGGCCG<br>TCAGTGCTGGACCCTCTTCT	107	13/14	0.1 µM	101%
TMEM45B	XM_021063152.1<br>XM_021063153.1<br>XM_021063154.1<br>XM_021063155.1	Sense<br>Antisense	TTGTGCTGGAGCTTTTCCGA<br>TTCTGGTCCCATTCAGGTGC	109	NA	0.2 µM	98%

	XM_021063156.1						
ENPEP	NM_214017.1	Sense<br>Antisense	TATCTGTGCAGCGGTAGTGG<br>GGAGATGTTCACTGTCCCCG	286	1	0.1 µM	102%
LOC110256649	XM_021072807.1	Sense<br>Antisense	ACAACACGGCTGAAGGTAGG<br>TCTGAGGTGGGATCAAAAGG	199	NA	0.2 µM	102%
CYP2J34	NM_001244633.1	Sense<br>Antisense	AGCGGTTTGACTACCAGGATG<br>AGCTGGACCTGCACTGATGTG	91	4/5	0.2 µM	100%
LOC100526118	XM_003128792.4	Sense<br>Antisense	GGTTATCCGGTGGCTTCTGG<br>TCTGGACCAACTTCATGCCA	152	NA	0.1 µM	101%
S100A2	XM_001929556.5	Sense<br>Antisense	GGTTGCCACCTTCCACAAGTA<br>CCAGGAAAACGGCATACTCC	203	NA	0.08 µM	102%
SPRY3	XM_013986552.2<br>XM_003135510.5	Sense<br>Antisense	CAGCATTACGCCCTCACCTT<br>GCTTCTCCCTTCAGAGCACC	96	NA	0.2 µM	100%
FOXG1	XM_021099188.1	Sense<br>Antisense	TATGAGAAGCCGCCGTTCAG<br>GAGGGACAGATTGTGGCGAA	171	NA	0.2 µM	100%
GTSF1	XM_003126205.6<br>XM_005663861.3	Sense<br>Antisense	AGCCCTGCAAGCAACATAGT<br>TGGCAGAACATACGGCAGAG	87	NA	0.2 µM	98%
SQLE	NM_001101026.1	Sense<br>Antisense	GCAGCTATTCTCCAGGCCAA<br>AGCGCAACTGGACCACTAAT	191	8/10	0.2 µM	99%
CHL1	XM_021069243.1<br>XM_021069244.1<br>XM_021069246.1<br>XM_013981901.2<br>XM_021069247.1<br>XM_021069248.1<br>XM_021069249.1<br>XM_021069250.1	Sense<br>Antisense	CTGCAAACAATGACCCTGTGT<br>GGAGGCAGCCCAGAAAGAAT	154	NA	0.2 µM	102%
UBA52	NM_214211.1	Sense<br>Antisense	CTGCCGCAAGTGTTATGCTC<br>TTTGACCTTCTTCTTGGGGCG	100	4/5	0.2 µM	102%

NA, Not Applicable

Gene symbol	Accession		Sequence	Amplicon length	Exon	[Primer]	Efficiency
***Homo sapiens* genes**							
PPP1R1B	NM_032192	Sense	GGGCACCATCTCAAGTCGAA	126	4/5	0.2 µM	92%
	NM_181505	Antisense	TGAGGCCTGGTTCTCATTCA				
AFP	NM_001134.3	Sense	CTTTGGGCTGCTCGCTATGA	176	5/6	0.2 µM	91%
	NM_001354717	Antisense	ATGGCTTGGAAAGTTCGGGT		6/7		
TMEM45B	NM_001331210.2	Sense	CTCTGTTCGGAGGGTGTGTT	158	5/6	0.2 µM	95%
	NM_138788.5	Antisense	GTCCCATTCGGGTGTTCCAA		4/5		
	NM_001331211.2				5/6		
	NM_001331212.2				5/6		
ENPEP	NM_001977.4	Sense	GAAGAGCTTACCCCCAGCAG	120	1	0.4 µM	100%
	NM_001379611.1	Antisense	GACTTGTCCGTTCTCCGTGT				
	NM_001379612.1						
	NM_001379613.1						
SPRY3	NM_005840.4	Sense	TGAACAGCTGCGCTCTACTC	237	2	0.4 µM	98%
	NM_001304990.2	Antisense	GCAGTGGTGCTATGGGACAT		3		
	NM_001394353.1				4		
	NM_001394354.1				4		
	NM_001394355.1				4		
SQLE	NM_003129.4	Sense	GCCTGCCTTTCATTGGCTTC	90	1/2	0.2 µM	99%
		Antisense	TTCCTTTTCTGCGCCTCCTG				
				132	4/5	0.2 µM	95%
GAPDH	NM_002046.7	Sense	CAAATTCCATGGCACCGTCA		3/4		
	NM_001256799.3	Antisense	GACTCCACGACGTACTCAGC		4/5		
	NM_001289745.3				3/4		
	NM_001289746.2						

Gene symbol	Accession		Sequence	Amplicon length	Exon	[Primer]	Efficiency
**Mouse genes**							
Ppp1r1b	NM_144828.2<br>NM_001313970.1	Sense<br>Antisense	CCCAACCCCTGTGCCTATAC<br>TCCCGAAGCTCCCCTAACTC	119	4/5	0.3 µM	90%
Afp	NM_007423.4	Sense<br>Antisense	AGTTTCCAGAACCTGCCGAG<br>ACCTTGTCGTACTGAGCAGC	145	4/5	0.1 µM	100%
Tmem45b	NM_144936.1	Sense<br>Antisense	TCGGGATAACATCGTGCTGG<br>GTCCCATTCTGGCCTTCCAA	115	4/5	100 µM	0.2%
Enpep	NM_007934.3	Sense<br>Antisense	GCAAACCCACTCGTGACCTA<br>CAGCCGATAGACACTGTCCC	188	1	0.2 µM	97%
Spry3	NM_001030293.3<br>NM_001401849.1<br>NM_001401850.1<br>NM_001401851.1	Sense<br>Antisense	GCTCTCCCACGCAGTATCAG<br>TTGATCAGAGGCAGTGGTGC	111	4	0.2 µM	98%
Sqle	NM_009270.3	Sense<br>Antisense	TACCTCAGTGTCGACCTCGT<br>TGGAGAGAACTGCTGCCAAG	92	2	0.2 µM	99%
Ppib	NM_011149.2	Sense<br>Antisense	GGAGATGGCACAGGAGGAA<br>GTAGTGCTTCAGCTTGAAGTTCTCAT	72	3/4	0.1 µM	103%
